# Chinese Cervicocephalic artery dissection study (CCADS): rationale and protocol for a multicenter prospective cohort study

**DOI:** 10.1186/s12883-018-1011-x

**Published:** 2018-01-11

**Authors:** Zhu Zhu, Yuyuan Xu, Yilong Wang, Zhenhua Zhou, Xiang Han, Aihua Liu, Jing Peng, Yi Xu, Luyao Wang

**Affiliations:** 1 Department of Neurology, State Key Laboratory of Medical Neurobiology, Huashan Hospital, Fudan University, Shanghai, China; 2 Department of Neurology, Beijing Tiantan Hospital, Capital Medical University, Beijing, China; 3 China National Clinical Research Center for Neurological Diseases, Beijing, China; 4 Center of Stroke, Beijing Institute for Brain Disorders, Beijing, China; 5 Beijing Key Laboratory of Translational Medicine for Cerebrovascular Disease, Beijing, China; 6 Department of Neurology, Southwest Hospital, Third Military Medical University, Chongqing, China; 7 Department of Interventional Neuroradiology, Beijing Tiantan Hospital, Capital Medical University, China; Beijing Neurosurgical Institute, Capital Medical University, Beijing, China

**Keywords:** Cervicocephalic artery dissection, Cohort, Risk factors, Magnetic resonance imaging, Biomarker, Prognosis

## Abstract

**Background:**

Cervicocephalic artery dissection (CAD) is an important etiology of stroke in the youth. Findings from recent studies suggest it a “group of disease entities” with different underlying etiologies, presentations and prognosis, necessitating an integral study including various types of CAD to get a better understanding of this disease. In addition, Chinese patients with CAD are likely to carry different features from their western counterparts, which remains uncertain yet. Chinese Cervicocephalic Artery Dissection Study (CCADS) therefore aims at exploring the epidemiology, risk factors, clinical/radiological features, diagnosis and prognosis of CAD in Chinese patients.

**Methods/design:**

CCADS is a multicenter prospective cohort study enrolling patients age ≥ 18 years with recent (<14 days after onset) CAD. Baseline clinical data, laboratory tests and imaging studies are performed within 3 days after admission, and follow-ups will be conducted through face-to-face interviews at discharge, 3 months, 6 months and 12 months after admission, when the modified Rankin Scale (mRS), cerebrovascular events, medication compliance, CAD evolution and so on are evaluated. Additional blood samples will also be collected at baseline, 3 and 12 months follow-up. The primary outcome is radiographic evolution of CAD; secondary outcomes include cerebrovascular events, major bleeding complications, all-cause mortality and functional independence.

**Discussion:**

Through the integration of information on epidemiology, risk factors, clinical/radiological features and prognosis of various types of CAD in Chinese population, combined with the application of advanced imaging techniques, collection of potential blood biomarkers, and assessment of novel treatment strategies. CCADS will provide thorough information on CAD - the major cause of stroke in the youth, and play a role in prevention and treatment determination in the future.

**Electronic supplementary material:**

The online version of this article (10.1186/s12883-018-1011-x) contains supplementary material, which is available to authorized users.

## Background

Artery dissection develops when blood enters the wall of a vessel and separates the layers, which can be caused by a tear in the intimal layer or rupture of the vaso vasorum in the media [1]. Cervicocephalic artery dissection (CAD) is an important etiology of stroke in the youth, accounting for 8%–25% of stroke events among patients aged 18 to 49 years [2, 3]. Thanks to the development of advanced imaging techniques, CAD has been increasingly recognized in the past few years, some aspects of this disease, however, remain mysterious to researchers.

Although CAD used to be considered a “single disease”, findings from recent studies suggest it a “group of disease entities” with various features based on different sites (intracranial vs extracranial, anterior vs posterior circulation), morphologies (intimal flap, double lumen or intramural hematoma), and presentations (isolated headache, cranial nerve palsies, ischemic stroke or subarachnoid hemorrhage) [4–6]. CADISP (Cervical Artery Dissection and Ischemic Stroke Patients) is the largest cohort study worldwide examining different profiles according to the dissection site in ischemic stroke patients with cervical artery dissection [7], while knowledge on either milder (asymptomatic) or more severe (intracranial artery dissection presenting as subarachnoid hemorrhage) type of CAD is lacking. Thus, to establish a cohort including various kinds of CAD may be helpful for identifying both commonness and individuality of this disease.

In addition, despite the overall recurrence rate of stroke induced by artery dissection being low [8, 9], the evolution of artery dissection itself remains uncertain, especially according to different treatments, underlying etiologies and clinical or morphological features. By assuming that the existence of CAD may worsen the vascular lesions in the long run particularly when atherosclerosis develops with aging, not only the clinical outcomes but also the radiographic evolution of CAD needs to be paid close attention to.

Finally, Chinese patients with cerebrovascular diseases have been reported to carry different profiles compared with their western counterparts [10]. The characteristics of CAD associated stroke in Chinese population, however, remain uncertain. Data from previous single-center research with relatively small sample size indicated higher proportion of intracranial artery dissection and posterior circulation involvement in Chinese CAD patients [11, 12]. These features, on one hand, make the diagnosis of CAD more challenging because of small lumen and tortuous course of involved vessels; on the other hand, carry high risk of hemorrhagic transformation when antithrombotic treatments are administered. At present, the diagnosis and management of CAD in China are largely based on specialist consensus due to lack of high-class evidence [13], thus necessitating the establishment of a Chinese cohort with large sample size for better clinical practice in the future.

Therefore, we describe here the protocol of CCADS, a multicenter prospective cohort study to explore the epidemiology, risk factors, clinical/radiological features, diagnosis and prognosis of CAD in Chinese patients incorporating the application of advanced imaging techniques, collection of potential blood biomarkers, and assessment of novel treatment strategies.

## Methods/design

### Study design

CCADS is a national, multicenter, consecutive, prospective, cohort study enrolling patients with cervical or intracranial artery dissection within 14 days after symptom onset. Patient recruitment will take place at 56 centers in China. Approval of the Ethics Committee at each center has been obtained and all participants or their next of kin provide written consent at the time of enrollment in the cohort. The total duration of the study will be approximately 4 years, from September 2017 (first in) until August 2021 (last out).

### Study objectives

The primary objective of this study is to get a better knowledge on epidemiology, risk factors, clinical presentations and prognosis of CAD in Chinese population by setting up a large cohort; to assess the effect of different treatment strategies on clinical and radiographic evolution of CAD. Secondary objectives include to: 1) find out the association between possible etiologies (e.g. hereditary diseases) or risk factors (e.g. infection) and characteristics of CAD (e.g. multiple dissections, dissecting aneurysm, etc.); 2) determine the relationship between baseline features, functional outcomes and radiographic evolution of CAD; 3) establish standardized process for early and accurate diagnosis of CAD by analyzing relatively specific clinical and radiological features; 4) investigate individualized therapies, laying a foundation for further clinical trials.

### Study population

All consecutive patients referred to the department of Neurology of 56 tertiary teaching hospitals and regional hospitals (see Additional file 1) in China with clinical or imaging work-ups suggesting CAD will be screened for eligibility. Patients fulfilling the inclusion and not the exclusion criteria are asked to participate.

Inclusion criteria:

1. Age 18 years or older.
2. Within 14 days after the first symptom onset for symptomatic patients; or asymptomatic patients.
3. Typical radiological characteristics in at least one confirmatory angiographic examination including magnetic resonance angiography (MRA), computerized tomographic angiography (CTA), or digital subtraction angiography(DSA): intimal flap, double lumen, dissecting aneurysm or luminal dilation plus stenosis. For an artery exhibiting non-specific stenosis or occlusion, CAD will be diagnosed if cross-sectional MRI demonstrated intramural hematoma and intraplaque hemorrhage is excluded [5, 14].

Exclusion criteria:

1. Pregnant women.
2. Patients refusing to participate the study.

### Procedures

**Fig. 1:**
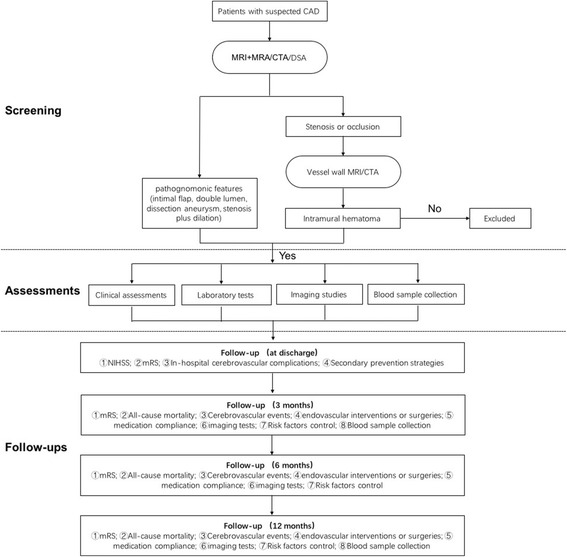
Flowchart of the study

Eligible patients will be recruited at the time of confirmed diagnosis of CAD. We intend to include 1300 patients, which is expected to complete over a 3-year period. Baseline assessments including clinical data, laboratory tests and imaging studies are performed within 3 days after admission, and a blood sample will be drawn from each patient during the time in hospital. Follow-ups will be conducted through face-to-face interviews at discharge, 3 months, 6 months and 12 months after admission, when the modified Rankin Scale (mRS), cerebrovascular events, medication compliance, CAD evolution and so on are going to be evaluated by trained neurologists. A flowchart of the study is shown in Fig. 1.

### Study measurements

#### Clinical data

Data of patients will be collected using an electronic Case Report Form (e-CRF) at baseline and during the follow-up period. Clinical items recorded include demographics, medical history, clinical presentations, physical examination findings and treatments in hospital. The detailed information is shown in Table 1.

**Table 1:** Clinical assessments for CAD patients in CCADS

Domain	Assessment
Demographics	age, gender, educational level, contact information
Medical history	
vascular risk factors	American Heart Association guideline [21]
migraine	classification and diagnostic criteria for headache disorders, cranial neuralgias and facial pain [22]
I CTD*	Ehlers-Danlos syndrome, Marfan Syndrome, Loeys-Dietz syndrome, etc. [23]
autoimmune diseases	SLE, Becet’s disease, Sjogren’s syndrome, etc.
mild trauma	neck manipulation or overextension (within 2 weeks prior to symptom onset) [24]
infection	within 2 weeks prior to symptom onset
Personal history	
smoking	a structured questionnaire embedded in CRF
alcohol use	a structured questionnaire embedded in CRF
risk factor control	hypertension, migraine, etc.
Clinical presentations	
stroke	clinical stroke syndrome (sudden neurological dysfunction lasting >24 h, with no apparent cause other than that of vascular origin)
TIA	rapidly evolving focal neurological deficit, without positive phenomena such as twitches, jerks or myoclonus, with no other than vascular cause lasting less than 24 h
cranial nerve palsy	peripheral hypoglossal nerve or facial nerve palsy
headache	new onset headache; severity, nature, location or frequency change
Horner syndrome	Miosis, partial ptosis, loss of hemifacial sweating
stroke severity	NIHSS [25]
stoke risk after TIA	ABCD2 scores [26]
Functional independence	mRS [27]
Treatment	
antithrombotic drugs	aspirin, clopidogrel, NOAC
reperfusion therapy	intravenous thrombolysis, intra-arterial thrombolysis, endovascular intervention
other medicines	statin, antihypertensive therapy, antidiabetics
medication compliance	a structured questionnaire
time	time from symptom onset to treatment, and treatment duration

*ICTD Inherited connective tissue disorders, *mRS* Modified Rankin Scale, *NIHSS* National Institute of Health Stroke Scale, *TIA* Transient ischemic attack, *NOAC* Novel oral anticoagulant, *SLE* Systemic lupus erythematosus

#### Laboratory tests and blood sample collection

Clinical biochemistry tests including total blood cell count, renal and liver functions, glucose and lipid levels, folic acid, Vitamin B12, homocysteine concentrations and autoimmune biomarkers will be measured using standard lab procedures within 3 days after admission and recorded at discharge of the patients.

Three additional blood samples (containing plasma, serum and white cells, respectively) will also be taken from the peripheral vein of each participant at baseline, 3 and 12 months follow-up. All the samples will be collected into tubes and stored at −20 °C until use for future pooled analysis.

### Imaging protocols

#### MRI

The MRI studies are performed on a 3 Tesla MR-scanner (DISCOVERY, MR750, GE Medical systems, Milwaukee or MAGNETOM Verio, SIEMENS Medical Systems, Germany). The scanning protocol include T1, T2, T2 fluid attenuated inversion recovery (FLAIR) and diffusion weighted imaging (DWI) sequences of the brain. Vascular MRI scanning is performed using standardized protocol consisting of magnetic resonance angiography (MRA) and high-resolution vessel wall imaging, which have been recommended for detection of CAD [6, 15, 16]. The corresponding sequences are described in Table 2.

**Table 2:** MRI parameters of vascular imaging

Scanner	Sequences	TR/TE (ms)	FOV (mm)	Matrix	Number of slices	Slice thickness(mm)	time
GE	3D TOF	25/3.4	220╳ 200	320╳192	96	1.4	3:32
	2D FSE	2500/85	150╳150	384╳256	12	2	2:45
	3D CUBE	350/15	200╳180	256╳192	32	1	2:46
SIEMENS	3D TOF	21/3.6	200╳180	256╳224	120	0.9	2:37
	2D FSE	1500/26	150╳150	256╳256	20	2	3:48
	3D SPACE	1500/252	200╳180	256╳224	56	0.8	4:14

*TR* Repetition time, *TE* Echo time, *FOV* Field of view, *Matrix* Frequency x phase, *TOF* Time of flight, *FSE* Fast spin echo

##### DSA

DSA is performed via transfemoral approach with intra-arterial injection of contrast medium, and all images are acquired on an Infinix (Toshiba Medical Systems, Tokyo, Japan) imaging system. Selective catheterization of the internal carotid arteries and the vertebral arteries are going to be done. Standard anteroposterior and lateral projection images, as well as magnified oblique projections, are obtained. For suspected artery dissection, rotational 3D angiography is also done to better delineate the details of the artery.

##### CTA

CTA is performed using 256-row CT scanners (Brilliance iCT, Royal Philips Electronics, NEDERLAND B.V.) with the scanning range from aortic arch to vertex (slice thickness: 0.9 mm; reconstruction interval: 0.7 mm; voltage: 100Kv; current: 125mAs; FOV: 220 mm). Ioxehol is injected from antecubital vein at a rate of 5 ml/s for a total of 50 ml in volume. Images will be transferred to post-processing workstation (Extended Brilliance Workspace, ver. 4.5, Philips) for further analysis and reconstruction. Maximum intensity projection (MIP), multiplaner reformations (MPR) and volume rendered (VR) techniques are applied to reconstruct raw image data. Both raw data and reconstructed models are used for evaluation.

### Image analysis and classification

Morphologies of CAD are grouped into intimal flap (a layer crossing the arterial lumen), double lumen (true plus false lumens), dissection aneurysm (luminal dilation), pearl-and-string sign (aneurysmal dilatation alternating with stenosis) and tapered steno-occlusion plus evidence of intramural hematoma (eccentric, intermediate-to-high signal intensity of the arterial wall according to hemorrhagic age). The proximal part (entry point) of a dissection lesion is defined as the site of artery dissection. As such, a dissection located completely within the cranium is classified as intracranial dissection, and a dissection starting extracranially as extracranial dissection. Multiple dissections will be recorded when more than two different arteries are involved; single dissection is recognized when the lesion localized in one vessel regardless of the length.

Two experienced neurologists will evaluate baseline and follow-up images independently at each center, and images will be reviewed centrally by 3 principle investigators (YL Wang, X Han, ZH Zhou) of the study in case of disagreement.

### Outcome measures

The primary outcome of this study is the radiographic evolution of CAD, which is classified as complete recovery, partial recovery, unchanged, or progression by comparing the radiological features of dissected arteries in follow-up period with those at baseline. Secondary outcomes include cerebrovascular events (ischemic or hemorrhagic stroke, TIA), major bleeding complications according to PLATO (Platelet Inhibition and Patient Outcomes) definition [17], all-cause mortality and functional independence assessed by mRS.

### Sample size and analysis

This is a cohort study without specific hypothesis concerning the primary outcome. So sample size is calculated based on the method recommended for registry study (10 times number of variable) [18], with the estimated number of variables as 110. The sample size is therefore of at least 1100, which is increased to 1300 considering possible losses.

Statistical analysis will be performed using SAS statistical package. Statistical significance for previously mentioned outcomes will be assessed using χ2 or Fisher’s exact test for categorical variables such as vascular risk factors, genders, treatment strategies, and t-test, ANOVA, Mann–Whitney U or Kruskal-Wallis test for continuous variables like age, NIHSS, etc. Logistic regression models will be applied to determine potential risk factors of CAD; Cox proportional hazards multivariate analysis will then be performed to identify clinical and radiological predictors of outcomes in patients with CAD, adjusted by variables with *P* < 0.1 on univariate analysis. The correlation between continuous variables will be tested with Spearman’s or Pearson’s coefficients, where appropriate. Two-tailed *P* < 0.05 is considered significant.

## Discussion

CCADS is a nationwide prospective study focusing on CAD – a probably underrecognized while definitely innegligible culprit of young stroke. The differences of this study from previous ones lie in:

First, both cervical and intracranial artery dissection will be included, with the latter type still far from being fully understood at present because of the very low prevalence worldwide [19, 20]. The findings of CCADS, therefore, may help get a better understanding of this disease. Similarly, the coverage of various types of artery dissection (anterior and posterior circulation, symptomatic and asymptomatic, stenosis/occlusion and dissecting aneurysm lesions) in combination with large sample size can provide comprehensive as well as characteristic information on CAD, hence laying foundation for future clinical trials on individualized management.

Second, in addition to cerebrovascular events and functional independence, the radiographic evolution of CAD will be also specially followed up, and the predictors associated with anatomic recovery or progression are going to be assessed in this study.

Third, additional blood samples will be collected at acute and chronic stages of CAD, the analysis of which may help find out potential biomarkers for early diagnosis or for mechanism investigations.

Finally, the large Chinese cohort carries both specific and common features of CAD, providing more thorough information on this important etiology leading to stroke in the youth, which will play a role in prevention and treatment determination in the future.

## Additional file

Additional file 1: CCADS collaborative group. Names of 56 centers participating the study and the corresponding principle investigator in each center. (DOCX 58 kb)

## Abbreviations

3D: Three dimensional
CAD: Cervicocephalic artery dissection
CADISP: Cervical artery dissection and ischemic stroke patients
CTA: Computed tomography angiography
DSA: Digital subtraction angiography
DWI: Diffusion weighted imaging
e-CRF: Electronic case report form
FLAIR: Fluid attenuated inversion recovery
FOV: Field of view
FSE: Fast spin echo
ICTD: Inherited connective tissue disorders
MIP: Maximum intensity projection
MPR: Multiplaner reformations
MRA: Magnetic resonance angiography
MRI: Magnetic resonance imaging
mRS: Modified Rankin scale
NIHSS: National institute of health stroke scale
NOAC: Novel oral anticoagulant
PLATO: Platelet inhibition and patient outcomes
SAH: Subarachnoid hemorrhage
SLE: Systemic lupus erythematosus
TE: Echo time
TIA: Transient ischemic attack
TOF: Time of flight
TR: Repetition time
VR: Volume rendered
