# Supplementary material for: Chinese Cervicocephalic artery dissection study (CCADS): rationale and protocol for a multicenter prospective cohort study
Source: BMC Neurol. 2018 Jan 11;18:6. doi: 10.1186/s12883-018-1011-x (PMC5765701; doi:10.1186/s12883-018-1011-x)
Supplement: Additional file 1: — CCADS collaborative group. Names of 56 centers participating the study and the corresponding principle investigator in each center. (DOCX 58 kb) [file 12883_2018_1011_MOESM1_ESM.docx]

**Additional file**

**CCADS collaborative group**

| **Recruiting Center** | **PI**  **(principal investigator)** |
| --- | --- |
| 1. Beijing Tiantan Hospital, Capital Medical University | Yilong Wang |
| 1. Huashan Hospital, Fudan University | Xiang Han |
| 1. Southwest Hospital, Third Military Medical University | Zhenhua Zhou |
| 1. South Branch of Anhui Provincial Hospital | Li Wang |
| 1. The First Hospital of Anhui Medical University | Yanghua Tian |
| 1. The First People's Hospital of Chenzhou | Zhe Tang |
| 1. General Hospital of Daqing Oil Field | Xuhai Gong |
| 1. Changhai Hospital, Second Military Medical University | Qinghai Huang |
| 1. Daping Hospital, Third Military Medical University | Meng Zhang |
| 1. The First Affiliated Hospital of Henan University of Science and Technology | Jisheng Qi |
| 1. Sanmenxia Central Hospital, Henan University of Science and Technology | Zhenyu Liu |
| 1. Qingyuan People's Hospital (The Sixth Affiliated Hospital of Guangzhou Medical University) | Xianglin Chen |
| 1. Guangdong Traditional Chinese Medicine Hospital | Jinsong You |
| 1. The Affiliated Hospital of Guangdong Medical University | Wangtao Zhong |
| 15. Guangxi People's Hospital | Lvli Li |
| 1. The first Affiliated Hospital of Guangxi Medical University | Chao Qin |
| 1. Affiliated Hospital of Guilin Medical University | Hao Li |
| 1. Naval General Hospital of PLA | Feng Qiu |
| 1. Handan Central Hospital | Juntao Li |
| 1. The Second Hospital of Hebei Medical University | Li Guo |
| 1. Jiangxi People's Hospital | Xinhui Qu |
| 1. The First Affiliated Hospital of Jinan University | Yusheng Zhang |
| 1. Second Affiliated Hospital , Kunming Medical University | Yongtao Yang |
| 1. Lishui People's Hospital | Yanan Tang |
| 1. Nanjing Gulou Hospital | Jingwei Li |
| 1. Nanjing General Hospital of Nanjing Military Command | Dezhi Liu |
| 1. Nanjing Second People's Hospital | Tong Li |
| 1. Affiliated Hospital of Nantong University | Yunfeng Zhang |
| 1. Ningbo Medical Treatment Center Lihuili Hospital | Yong Chen |
| 1. Tianjin Medical University General Hospital | Ming Zou |
| 1. General Hospital, Ningxia Medical Universiry | Binwu Ma |
| 1. Puyang People's Hospital | Shunqing Zhang |
| 1. The Second Hospital of Shandong University | Zhengyu Zhu |
| 1. Qilu Hospital of Shandong University (Qingdao) | Haifeng Li |
| 35. Yantai Yuhuangding Hospital | Zhigang Liang |
| 1. Shanghai First People's Hospital, Shanghai Jiao Tong University | Yuncheng Wu |
| 1. General Hospital of Shenyang Military Area Command | Huisheng Chen |
| 1. The Eighth Hospital of Shenzhen | Guode Wu |
| 1. Shenzhen Second Hospital (The First Affiliated Hospital of Shenzhen University) | Lijie Ren |
| 1. Xuanwu hospital, Capital Medical University | Haiqing Song |
| 1. JiangSu Province SuBei Hospital, First Affiliated Hospital of Yangzhou University | Jun Xu |
| 1. JiangSu Province SuBei Hospital, First Affiliated Hospital of Yangzhou University | Yongjun Cao |
| 1. Taizhou First People's Hospital, Huangyan Hospital of Wenzhou Medical University | Peng Wang |
| 1. Tianjin Huanhu Hospital | Wei Yue |
| 1. Yijishan Hospital of Wannan Medical University | Xiangjun Xu |
| 46 The First Affiliated Hospital of Wenzhou Medical University | Beilei Zhu |
| 1. Zhongnan Hospital of Wuhan University | Yumin Liu |
| 1. Tongji Hospital of Tongji Medical University, Huazhong University of Science and Technology | Xiang Luo |
| 1. Yixing People's Hospital | Junfeng Shi |
| 1. Yixing People's Hospital | Min Lou |
| 1. The First Affiliated Hospital, Zhejiang University | Ziqi Xu |
| 1. Zhejiang Quhua Hospital | Yi Tan |
| 1. The First Hospital of China Medical University | Chuansheng Zhao |
| 1. The Second Affiliated Hospital, Sun Yat-sen (Zhongshan) University | Yongteng Xu |
| 1. The First Affiliated Hospital, Sun Yat-sen (Zhongshan) University | Yuhua Fan |
| 1. Zhongshan Hospital, Fudan University | Peng Du |
